# Role of SrtA in Pathogenicity of *Staphylococcus lugdunensis*

**DOI:** 10.3390/microorganisms8121975

**Published:** 2020-12-11

**Authors:** Muzaffar Hussain, Christian Kohler, Karsten Becker

**Affiliations:** 1 Institute of Medical Microbiology, University Hospital Münster, 48149 Münster, Germany; muzaffa@uni-muenster.de; 2 Friedrich Loeffler-Institute of Medical Microbiology, University Medicine Greifswald, 17475 Greifswald, Germany; christian.kohler@med.uni-greifswald.de

**Keywords:** *Staphylococcus lugdunensis*, sortase A, surface proteins, LPXTG

## Abstract

Among coagulase-negative staphylococci (CoNS), *Staphylococcus lugdunensis* has a special position as causative agent of aggressive courses of infectious endocarditis (IE) more reminiscent of IEs caused by *Staphylococcus aureus* than those by CoNS. To initiate colonization and invasion, bacterial cell surface proteins are required; however, only little is known about adhesion of *S. lugdunensis* to biotic surfaces. Cell surface proteins containing the LPXTG anchor motif are covalently attached to the cell wall by sortases. Here, we report the functionality of *Staphylococcus lugdunensis* sortase A (SrtA) to link LPXTG substrates to the cell wall. To determine the role of SrtA dependent surface proteins in biofilm formation and binding eukaryotic cells, we generated SrtA-deficient mutants (Δ*srtA*). These mutants formed a smaller amount of biofilm and bound less to immobilized fibronectin, fibrinogen, and vitronectin. Furthermore, SrtA absence affected the gene expression of two different adhesins on transcription level. Surprisingly, we found no decreased adherence and invasion in human cell lines, probably caused by the upregulation of further adhesins in Δ*srtA* mutant strains. In conclusion, the functionality of *S. lugdunensis* SrtA in anchoring LPXTG substrates to the cell wall let us define it as the pathogen’s housekeeping sortase.

## 1. Introduction

As known for *Staphylococcus aureus* and several coagulase-negative staphylococci (CoNS), *Staphylococcus lugdunensis* is also part of the human microbiota colonizing miscellaneous skin surface habitats [1,2,3,4]. Infections due to this opportunistic pathogen resemble those caused by *S. aureus* rather than “classical” CoNS infections [5]. In particular, aggressive and highly destructive courses of native and prosthetic valve infectious endocarditis (IE) have been reported [6]. Despite its clinical impact, only a few factors contributing to the pathogenicity of *S. lugdunensis* have been described, including a fibrinogen binding protein (Fbl), a von Willebrand-factor binding protein (vWbl), and a multifunctional autolysin (AtlL) [7,8,9,10,11]. 

To initiate invasive infections, staphylococcal cells irrespective of the species must adhere to cells of the host tissue or to the extracellular matrix (ECM) [12]. This complex multifactorial process is mediated by strong interactions of cell wall-anchored (CWA) proteins with host structures. In *S. aureus*, four distinct categories of CWA proteins have been identified, and the microbial surface components recognizing adhesive matrix molecules (MSCRAMMs) were elucidated as the largest group [13,14]. CWA proteins are characterized by a sorting signal containing a carboxy-terminal LPXTG anchor motif [15]. In *S. aureus* and many other Gram-positive species, the LPXTG-sorting signal is cleaved by the membrane-bound transpeptidase sortase. Sortase A (SrtA) of *S. aureus* is a 206 amino acid peptide that catalyzes a transpeptidation process [16,17,18,19] and is considered the housekeeping sortase [20]. The cleavage between the threonine and glycine residues is followed by an amide linkage between the carboxyl group of threonine and the amino group of a pentaglycine cross-bridge of the cell membrane-attached peptidoglycan [16,19]. Mutants lacking the *srtA* gene are defective in the cell wall anchoring of LPXTG proteins [16,17,21]. Thus, cells are unable to anchor cell surface proteins of *S. aureus* important for the adherence to eukaryotic cell structures. In consequence, Δ*srtA* mutants were attenuated to establish infections as shown in a murine model [22]. In addition to SrtA, a further *S. aureus* sortase isoform, sortase B (SrtB), has been described [23]. More recently, a SrtA-deficient mutant of *S. lugdunensis* was significantly less virulent than the parental strain in a rat IE model [24]. Therefore, the use of SrtA inhibitors might represent a promising anti-virulence therapy strategy to disrupt the anchoring of bacterial surface proteins, which are critical for the pathogen´s adherence to the host.

Here, we report the functionality of *S. lugdunensis* SrtA to anchor LPXTG substrates to the cell wall and defined it as the pathogen´s housekeeping sortase. Generating *srtA*-deficient mutants, we determined the role of *S. lugdunensis* SrtA-dependent surface proteins in biofilm formation and invasion of eukaryotic cells. Finally, we confirmed the influence of a functional SrtA on the gene expression of further LPXTG proteins.

## 2. Materials and Methods

### 2.1. Bacterial Strains and Culture Media

Lysogeny broth (LB) or agar were used for cultivation of *Escherichia coli* and staphylococci were cultivated in tryptic soy broth (TSB) or agar (TSA) (Difco, Detroit, Detroit, MI, USA), brain heart infusion (BHI) broth or agar (Merck, Darmstadt, Darmstadt, Germany) and Mueller Hinton (MH) broth or agar (Mast, Merseyside, Bootle, UK). Antibiotics were added to MH agar in appropriate amounts (ampicillin, 100 µg/mL, Sigma; erythromycin, 10 µg/mL Serva; and chloramphenicol, 10 µg/mL, Serva, Heidelberg, Germany) for selection of resistance in *E. coli* or *S. lugdunensis*. All bacterial strains and plasmids used in this study are presented in Table 1.

### 2.2. Growth Characteristics

Bacteria were grown in BHI overnight followed by dilution of the culture in 100 mL fresh BHI in 500 mL flask to an optical density 578 nm (OD_578_) of 0.05. Flasks were incubated at 160 rpm at 37 °C and the OD_578_ were determined hourly for a period of 10 h followed by final sampling after 24 h to establish growth curves. For growth experiments in little volumes, bacteria were grown in 5 mL BHI in a glass tube for 18 h at 37 °C with shaking at 160 rpm. Next day, OD_578_ were measured against un-inoculated BHI after vortex of growth. 

### 2.3. Characterization of Agglutination

The Pastorex Staph Plus (Sanofi Diagnostic Pasteur, S.A., Marnes la Coquette, France), a rapid agglutination test for the simultaneous detection of the *S. aureus* fibrinogen affinity antigen (clumping factor), protein A, and capsular polysaccharides were used to differentiate wild type and mutant strains.

### 2.4. Biofilm Formation

Bacteria were grown in 5 mL BHI, TSB, BHI + 0.5% glucose or TSB + 0.5% glucose in glass test tubes for 8 h at 37 °C with shaking at 160 rpm. Afterwards, the cells were diluted 1:100 with the same type of fresh medium and 100 µL were added to wells in quadruplicate in a 96 well microtiterplate. Plates were incubated at 37 °C without shaking overnight. Next day bacteria were removed, plates were washed with PBS, and the biofilm bacteria mix were fixed with ice cold methanol for 10 min at −20 °C. After washing once with PBS, the adhered biofilm bacteria were stained with crystal violet for 10 min at room temperature. Excess stain was removed by washing 3x with PBS. The adhered bacteria were brought into solution by the addition of 100 µL of 35% acetic acid to each well. Finally, the plates were read at OD_595_ in the iMark Microplate Reader (Bio Rad).

### 2.5. DNA Manipulations and Transformations

Staphylococcal cells were lysed with recombinant lysostaphin (20 U/mL, Applied Micro, New York, NY, USA). Genomic DNA isolated by using QIAamp DNA Mini Blood Kit (Qiagen, Hilden, Germany) and plasmid DNA were prepared using the Qiagen Plasmid Mini kit (Qiagen). DNA fragments were isolated from agarose gels using the QIAEX II Gel extraction kit (Qiagen). 

### 2.6. Construction of a srtA-Deficient Mutant

The method for the generation of a *srtA* lacking mutant was same as described before [7]. In brief, a primer set 1 (SrtA1FH, and SrtA1RE,) and primer set 2 (SrtA2FE, and SrtAl2RB,) were used to amplify PCR products of 809 and 793 bp, respectively, from genomic DNA of *S. lugdunensis* strain Sl48. The PCR products were ligated into the shuttle vector pBT9 and the ligation mixes were transformed into *E. coli* TG1 cells followed by incubation on LB plates containing ampicillin. A clone containing *srtA* gene as an insert was designated as pBT*srtA*. The restriction analysis and sequence data of clone pBT*srtA* confirmed *srtA* gene as an insert. A 1,479 bp *ermB* fragment was PCR-amplified using primers ermbF, and ermbR from the plasmid pEC4 containing the staphylococcal transposon Tn551 in a ClaI restriction site [28]. The *ermB* primers were designed from NCBI accession # AF239773. The *ermB* cassette was restricted with EcoRI and ligated into the EcoRI-restricted pBT*srtA*. The freshly prepared *E. coli* TG1 cells were transformed with the ligation mix and cultivated on ampicillin- and erythromycin-containing LB plates. Clones with the plasmid conferring resistance to both antibiotics were designated as pBT*srtA*E and were selected for further work. For the construction of the *srtA* allelic replacement mutant, protoplasts of *S. lugdunensis* strains Sl44 and Sl48 transformed with pBT*srtA*E as described by Palma et al. [29]. Chloramphenicol-sensitive and erythromycin-resistant colonies were detected by replica plating protocol onto plates containing chloramphenicol or erythromycin at 37 °C overnight. Clones that were sensitive for chloramphenicol and resistant to erythromycin were designated Mut7 (wild-type strain Sl44) or Mut47 (Wild-type strain Sl48) and were selected for further analyses. Correct insertion of the *ermB* was confirmed by using srtA1FH, srtA2RB, ermBF and ermBR primers.

### 2.7. The Complementation of a srtA-Deficient Mutant

A PCR product of *srtA* gene including the ribosomal binding site was PCR amplified using primer set (SrtA1FB and SrtA2RH) from genomic DNA of *S. lugdunensis* Sl48. The PCR product was restricted with HindIII and BamHI and ligated into the pCU1 plasmid. Freshly prepared *E. coli* TG1 cells were transformed with ligation mix. A plasmid containing *srtA* as an insert was designated pCU*srtA*. The isolated plasmid pCU*srtA* transformed by protoplast method into Mut7 and Mut47. Transformants were grown on TSA plates containing 10 µg of chloramphenicol and 10 µg of erythromycin per mL. Clones expressing SrtA were designated as Mut7C and Mut47C.

### 2.8. Cell Protein Preparations

Briefly, bacteria were grown overnight in BHI at 37 °C with shaking (160 rpm) and cells were harvested by centrifugation and washed twice with phosphate buffered saline. Then the cell surface proteins from PBS washed bacterial cells pellets were extracted by following methods: In the first method, bacterial cell surface proteins were generated by the LiCl method as described earlier [30]. In a second method we used the hydroxylamine hydrochloride method as described by Ton-That et al. [17]. In brief, bacteria were grown in two tubes containing 5 mL BHI overnight at 37 °C and 160 rpm. After centrifugation of the cells (4000 rpm 4 °C for 10 min), 1.5 mL of 50 mM Tris HCl pH 7.0 was added to the control pellet. To the other pellet we added 1.5 mL 100 mM hydroxylamine HCl in 50 mM Tris HCl pH 7.0. Both sets were stirred at 600 rpm at 37 °C in a heating block for 1 h. After centrifugation of the cells (13,000 rpm 4 °C for 10 min, the proteins in both supernatants were precipitated by addition of TCA to a final concentration of 10%. After centrifugation of the precipitated proteins (13,000 rpm 4 °C for 10 min), 200 µL of a 2× standard Laemmli SDS Page Sample buffer were added to the pellets and heated at 95 °C for 3 min. Samples were ready to load on SDS page gel. Whole cell lysates were prepared by the suspensions of cell pellets solved in 50 mM Tris/HCl pH 8.0 containing 15 mM NaCl. Lysostaphin (20 U/mL) and protease inhibitor cocktail (Sigma) were added and the mixture was incubated at 37 °C for 30 min. Then DNase was added to break DNA and, after centrifugation, liquid supernatants were used as whole cell lysate. For the generation of cell wall, cytoplasmic, and membrane proteins, *S. lugdunensis* were grown overnight in BHI at 37 °C with shaking (160 rpm) and cells were harvested by centrifugation and washed twice with phosphate buffered saline, and adjusted to an optical density at 600 nm (OD_600_) of 20. A 1-mL portion of the bacterial suspension was pelleted and resuspended in 200 μL of digestion buffer (50 mM Tris-HCl, 20 mM MgCl_2_, 30% [wt/vol] raffinose; pH 7.5) containing complete mini-EDTA-free protease inhibitors (Roche). Cell wall proteins were solubilized by digestion with lysostaphin (500 μg/mL) at 37 °C for 30 min. Protoplasts were harvested by centrifugation (5000× *g*, 15 min) and the supernatant was retained as the cell wall fraction. Protoplast pellets were washed once in digestion buffer, sedimented (5000× *g*, 15 min), and resuspended in ice-cold lysis buffer (50 mM Tris-HCl [pH 7.5]) containing protease inhibitors and DNase (80 μg/mL). Protoplasts were lysed on ice by vortexing. Complete lysis was confirmed by phase-contrast microscopy. The membrane fractions were obtained by centrifugation at 18,500× *g* for 1 h at 4 °C. The pellets (membrane fraction) were washed once and resuspended in ice-cold lysis buffer. Cell wall fractions and protoplast suspensions were centrifuged under the same conditions and the pellets were resuspended in 200 μL of lysis buffer. The liquid supernatant from protoplast suspension retained as cytosolic fraction.

### 2.9. SDS-PAGE and Ligand Overlay Analysis

The prepared cell surface proteins were separated in SDS-PAGE mini gel approaches. For Western ligand blot analysis, fibronectin (Fn) (Chemicon, Temecula, CA, USA), fibrinogen (Fg) (Calbiochem, San Diego, CA, USA), collagen type I (Cn) (Sigma; Sigma product #7774) or vitronectin (Vn) were purified by the method of Yatohgo et al. [28] and labeled with biotin (Roche, Mannheim, Germany). The cell surface proteins separated by SDS-PAGE were transferred electrophoretically (Trans-blot SD, Bio-Rad, Munich, Germany) onto nitrocellulose membranes (Schleicher and Schüll, Dassel, Germany) and were blocked with 3% BSA (bovine serum albumin fraction V, Sigma). The nitrocellulose membranes with blotted proteins were exposed to biotinylated ligands, treated with avidin alkaline phosphatase and subsequently bands were revealed using NBT (Nitrotetrazolium Blue chloride) and BCIP (5-Brom-4-chlor-3-indoxylphosphat) as recommended by the manufacturer´s protocol (Bio-Rad).

### 2.10. Expression of Recombinant Sortase-A

The *srtA* gene was PCR amplified from genomic DNA of *S. lugdunensis* using the primer set srtAF and srtAR. The *srtA* gene was ligated into the pQE30 expression vector and the vector was transformed into *E. coli* TG1 cells. *E. coli* TG1 cells were cultivated in LB medium plus 100 µg/mL ampicillin at 37 °C and 150 rpm and IPTG (Isopropyl-β-D-thiogalactopyranosid, Sigma) 1 mM per mL was added at OD_578_ 0.5 to induce the expression of SrtA. *E. coli* cells were lysed by lysozyme (1 mg/mL lysis buffer) and SrtA was purified in a single step under native conditions using Ni-nitrilotriacetic acid (NINTA) resin column according to the manufacturer’s recommendation (Qiagen).

### 2.11. Polyclonal Antibodies

Polyclonal antibodies against the recombinant SrtA were raised commercially (Genosphere Biotechnologies, Paris, France) in two rabbits by applying standard procedures of 70 days with 4 immunizations. The IgG fraction from the crude antiserum was obtained on protein-A column (Pierce, Rockford, IL, USA). In IgG preparations, naturally occurring anti-staphylococcal antibodies were removed by mixing the sera with 10 volumes of LiCl cell surface extracts from strain Mut47, which does not produce the SrtA, and immune-complexes were partially removed by centrifugation at 14,000 rpm at 4 °C for 15 min. For control Western immunoblotting, cell proteins of the wild type were separated on a SDS-Page, transferred onto a nitrocellulose membrane (Schleicher and Schuell, Dassel, Germany), and probed with anti-SrtA sera. Bound rabbit immunoglobulin G was detected with alkaline phosphatase-conjugated goat anti-rabbit immunoglobulins (Dako Diagnostika, Hamburg, Germany) with NBT/BCIP color reaction (Bio-Rad). 

### 2.12. ELISA Adherence assays

The employed ELISA adherence assays of staphylococci to ECM proteins and host cells were done as described earlier [7]. 

### 2.13. Cell Culture and Flow Cytometric Invasion Assay

For flow cytometric invasion assay, three types of host cells were used (Table 1). The human endothelial cell line, Ea.hy926 [31], was grown in DMEM (Sigma-Aldrich) supplemented with 10% fetal calf serum (FCS).The fibroblasts (human fetal lung cells) were cultivated as described earlier [31,32]. Another epithelial cell line, the human bladder carcinoma cell line 5637 (ATCC HTB-9™), which secretes several functionally active cytokines, was also used in this study and maintained as described by Quentmeier et al. [33]. Fluorescein-5-isothiocyanate (FITC)-labeling of bacterial cells was performed as described elsewhere [34,35] and labeled cells were used within 24 h. The flow cytometric invasion assay was performed as described previously with minor modifications, such as the addition of propidium iodide just before samples were analyzed on a FacsCALIBUR™ (BD Bioscience) [36]. Results were normalized according to the mean fluorescence intensity of the respective bacterial preparation, as determined by flow cytometry. The invasiveness of the laboratory *S. aureus* strain Cowan I was set as 100% and the results are shown as means ± SEM of three independent experiments performed in duplicates. 

### 2.14. Sortase Inhibitor PVS

5 mL BHI in a glass tube was inoculate with *S. lugdunensis* and grown overnight at 37 °C with 160 rpm. Next day, cultures were diluted to an OD_595_ of 0.01. 180 µL of diluted culture was mixed with 20 µL of 10× concentrated phenyl vinyl sulfone (PVS) in a microtiter plate well. Plates were incubated at 37 °C for 18 h. Growth was determined in a Biorad reader at absorbance 595 nm. The lowest concentration that inhibited the cell growth was considered to be the MIC. For determination of effects of PVS on binding of bacteria to Fn and Fg, microtiter plate wells were coated with Fn and Fg separately. Overnight cultures were diluted to an OD_595_ of 0.01. 180 µL of diluted culture were mixed with 20 µL of concentrated phenyl vinyl sulfone (PVS) to the respective concentrations in a microtiter plate well. Plates were incubated at room temperature for 1 h. Adherence was determined in a Biorad reader at absorbance of 595 nm.

### 2.15. Hydroxylaminolysis of LPXTG Peptide

Reactions were performed in 260 μL volume containing 50 mM Hepes buffer pH 7.5, 5 mM CaCl_2_, and 10 μM LPETG fluorescent labeled peptides (DABCYL-LPETG-EDANS). The mixture was heated at 95 °C for 5 min. Then 10 µL of staphylococcal LiCl-extracts or 10 μM recombinant sortase-A, 5 µM of the sortase inhibitor p-hydroxymecuribenzoic acid (pHMB), or 10 mM DTT were added. Reactions were incubated for 1 h at 37 °C and then analyzed fluorometrically at 350 nm for excitations and 495 nm for recordings. Experiments were performed in triplicates.

### 2.16. Real-Time Reverse-Transcription PCR (qtRT-PCR)

For RNA isolation, *S. lugdunensis* was grown in BHI for 18 h at 37 °C with 160 rpm. After bacterial RNA isolation, real-time amplification and transcripts quantification was done as described earlier [37]. Primer sequences are given in Table 2.

### 2.17. Statistical Analysis

Using GraphPad Instat3, the statistical significance of the results was analyzed by ANOVA in combination with Bonferroni´s post test (compare all pairs of column or compare selected pairs of column) or Dunnett´s post test (compare all columns vs. control). Differences with *p* values ≤ 0.05 were considered as significant and are indicated with asterisks: * (*p* ≤ 0.05), ** (*p* ≤ 0.01), and *** (*p* ≤ 0.001).

## 3. Results

### 3.1. Sortase A-Dependent Proteins

A comparative bioinformatic analysis of publicly available *S. lugdunensis* genomes identified the presence of only 11 sortase-A dependent proteins (Table 3) [39]. All 11 MSCRAMMs were found to be highly conserved in the *S. lugdunensis* strains as genomic data from strain HKU09-01, M23590, VCU139 and N920143 revealed [39]. However, there exist only minor differences in the number of repeats within the stalk regions that in turns affect the length of mature proteins [39]. 

### 3.2. Alignment of Sortase A Sequences

**Figure 1:**
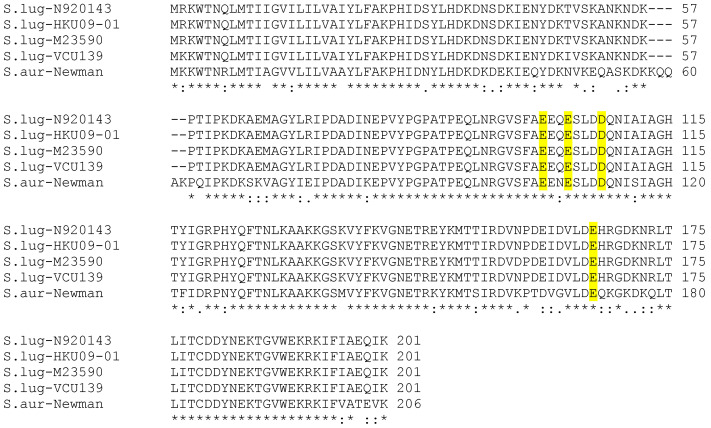
Multiple sequence alignments of SrtA sequences of four strains of *S. lugdunensis* (N920143, HKU09-01, M23590, and VCU139) and *S. aureus* Newman using CLUSTAL 2.1. The highly conserved amino acids glutamate (E) and aspartate (D) involved in binding of calcium ions are highlighted in yellow. Stars denote highly conserved amino acids.

In silico analyses identified homologs of *S. aureus srtA* in published genomes of *S. lugdunensis*. A nucleotide NCBI BLAST search identified SLGD_00472 (strain HKU09-01), *srtA* (strain N920143 and M23590), and SEVCU139_1681 (strain VCU139) as homologs of staphylococcal *srtA*. A ClustalW alignment of amino acid sequences was performed for pairwise comparison of SrtA between the four *S. lugdunensis* strains (N920143, HKU09-01, M23590, VCU139) and *S. aureus* SrtA. The SrtA sequences of four strains of *S. lugdunensis* (N920143, HKU09-01, M23590, VCU139) showed an intra-alignment score of 98–100%. The identity of SrtA sequences from four *S. lugdunensis* strains to *S. aureus* Newman was between 76–77% and revealed significant similarities. We identified conserved amino acid residues in the region corresponding to the calcium binding cleft of the *S. aureus* SrtA, such as the three glutamate residues Glu105, Glu108 and Glu166 as well as the aspartate residue Asp112, which is also highly conserved in the SrtA sequences of the *S. lugdunensis* strains (Figure 1) [40].

### 3.3. Generation of Sortase-A Mutants

**Figure 2:**
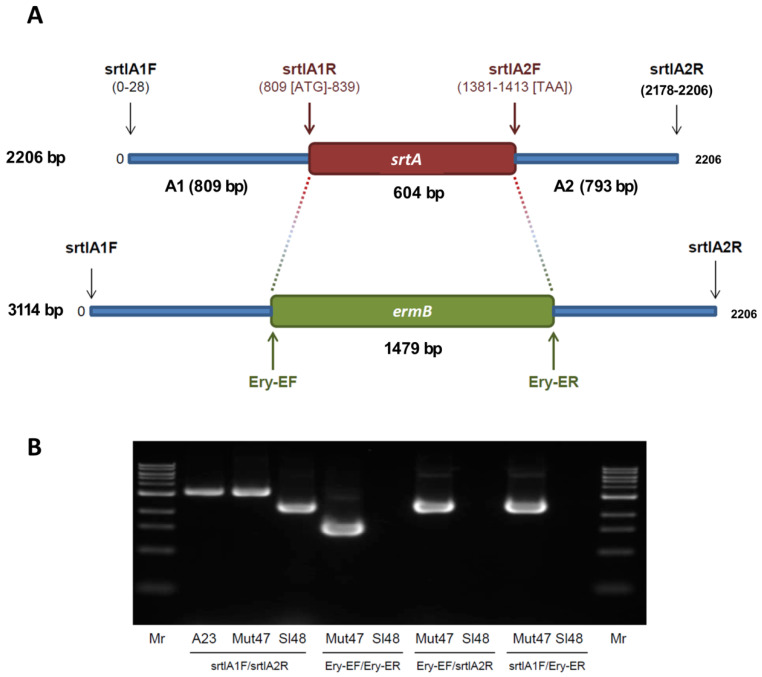
Genetic map of srtA and primer positions for PCRs, and results of PCR confirming the gene depletion of *srtA* in *S. lugdunensis*. (**A**) The gene loci of *srtA* in *S. lugdunensis* are shown before and after the recombination processes. In addition, the gene loci of *srtA* in *S. lugdunensis* are shown before and after the recombination processes and the positions of primer binding during the PCR experiments. The primer sequences are given in Table 3. (**B**) PCR products from genomic DNA of *S. lugdunensis* wild-type strain (Sl48), Δ*srtA* mutant strain (Mut47) and plasmid DNA of pBT*srtA* (A23) were separated by 1% agarose gels. Higher molecular bands (PCR with primer pair srtlA1F and srtlA2R) and the occurrence of corresponding bands to PCR experiments with primer pairs Ery-EF/Ery-ER, Ery-EF/srtlA2R and srtlA1F/Ery-ER confirming the deletion of *srtA* and following substitution by the *ermB* gene in the genome *S. lugdunensis* Δ*srtA* mutant strain Mut47.

To generate knockout mutants of *srtA*, we used the homologous recombination method leading to an insertion of *ermB* cassette into the *srtA* genes of *S. lugdunensis* strains Sl48 and Sl44 (Figure 2A). The *srtA* deficient mutants Mut7 and Mut47 were analyzed by PCR amplification using primers that anneal to *ermB* or to sequences flanking the *srtA* gene. The *ermB* gene could be amplified from the chromosomal DNA of the *srtA* deficient mutants Mut7 and Mut47, but not from the isogenic parent strains Sl48 and Sl44 respectively. Amplifications with primers specific for sequences flanking *srtA* revealed the insertion of *ermB* into the *srtA* genes of strains Mut7 and Mut47. The final double cross-over *srtA*::*ermB* allelic replacement in the Δ*srtA* mutant was confirmed by PCR amplification. Figure 2 shows the map of *srtA* as well as the positions of the primer pairs and the expected sizes of the amplified PCR products. The expected PCR product size with primers SrtA1F and SrtA2R was 2.2 Kb for the wild type and is 3.1 kb for the mutant. The observed sizes for the increased PCR products could only be generated from the Δ*srtA* mutants with primers SrtA1F and SrtA2R, but not with DNA of the wild type strains. In addition, amplifications of the *ermB* cassette were only successful with the Δ*srtA* mutant, but not with wild type strain DNAs using primers Ery-FE and Ery-RE which confirmed the allelic replacement of the wild type *srtA* gene by *srtA*::*ermB* (Figure 2). Results are exemplary shown for the Sl48 parental and Mut47 mutant strain. 

### 3.4. Characterisation of the Surface Proteins by SDS Page and Western Blot Experiments

**Figure 3:**
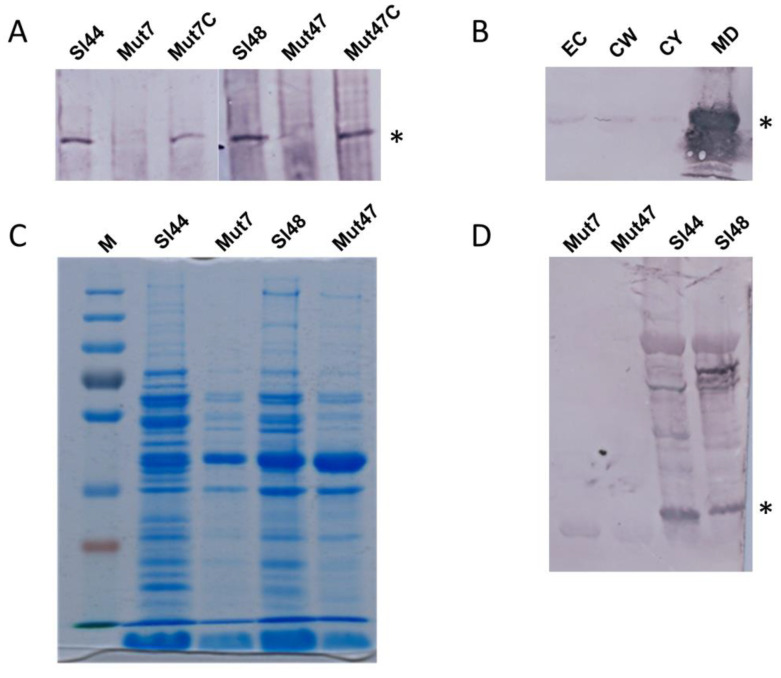
Western blot and SDS page experiments of *S. lugdunensis* wild type, Δ*srtA* mutant and complemented mutant strains showing the presence and localization of SrtA and consequences of a *srtA* deletion for the surface proteome pattern. (**A**) Western blots showing the absence of SrtA in the mutant strains but specific signals in wild types and complemented mutants. Whole cell extracts of wild type, mutant and complemented mutant strains were separated via SDS page and blotted. The Western blots were probed with specific polyclonal antibodies rose in rabbits against recombinant SrtA (anti-SrtA). Asterisk mark the specific SrtA band. (**B**) Localization of SrtA within *S. lugdunensis* cells. For this purpose, *S. lugdunensis* were grown in BHI broth medium. Cells were fractionated into the extracellular fraction (EC), cell wall fraction (CW), cytosol fraction (CY), and membrane digest fraction (MD). Proteins were separated by SDS page. After blotting, SrtA was detected in the different fractions with anti-SrtA antibodies. Asterisk mark the specific SrtA band. (**C**) Analyses of cell surface proteins of the wild-type strains and the mutant strains. The cell surface proteins were extracted in 0.1 M hydroxylamine hydrochloride. The protein extracts were separated on SDS-Page and stained with Coomassie Blue. (**D**) Cell surface protein fractions of wild type and mutant strains were separated via SDS page. After blotting, the Western blots were probed with a mixture of antibodies raised in rabbits against formaldehyde fixed whole cells of *S. lugdunensis*. Asterisk mark the specific SrtA band. M—page ruler, Sl44 and Sl48—wild type strains, Mut7 and Mut47—Δ*srtA* mutant strains, Mut7C and Mut47C—complemented Δ*srtA* mutant strains.

First, we verified the absence of the sortase A protein in mutant strains and confirmed their complementation with plasmid coded *srtA*. Specific polyclonal antibodies rose in rabbits against recombinant sortase-A lacking the N-terminal signal peptide, revealed sortase-A as a polypeptide of 26 kDa in staphylococcal extracts. Immunoblotting with anti-SrtA antibodies failed to detect the SrtA in the whole cell extract from mutant strains, but complementation of the mutant strain with plasmid pCUSrtA encoded sortase-A restored the appearance of the sortase-A (Figure 3A). As a control, the transformation of the mutant strain with empty vector DNA did not affect the expression of the SrtA (data not shown). Together, these results confirmed the successful depletion of the *srtA* gene from the genome of *S. lugdunensis*. The SrtA is described as a membrane bound protein. Therefore, an attempt was made to localize SrtA within *S. lugdunensis* cells. For this purpose, *S. lugdunensis* cultivated in BHI broth medium was fractionated into the extracellular fraction, cell wall fraction, cytosol fraction and membrane digest. SrtA was detected in the membrane digest (Figure 3B). Strongest signal was observed for the membrane fractions. Only weak signals were found for the other protein fractions. It shows that SrtA of *S. lugdunensis* is also a membrane bound protein.

### 3.5. The Surface Proteomes of the Wild-Type and Mutant Strains Differ in the Absence of the SrtA

The cell surface protein expression patterns were determined by extracting the whole cells in 0.1 M hydroxylamine hydrochloride. In Coomassie Blue-stained SDS-PAGEs, the wild type and the mutant strains showed similar but non-identical patterns of proteins. In general, gels revealed less protein concentrations and protein bands for the mutants strains compared to the wild type isolates. In all strains, lower molecular mass proteins were detected in the both wild type and mutant strains, but higher molecular mass proteins were dominant in the wild type strains (Figure 3C). The results were confirmed by Western immunoblots probed with a mixture of antibodies raised in rabbits against formaldehyde fixed whole cells of *S. lugdunensis*. This experiment revealed a lack of bands for several proteins of the cell surface protein extracts of the Δ*srtA* mutant strains, although these bands were present in wild type strains (Figure 3D).

### 3.6. Determination of Growth and Biofilm Formation of the ΔsrtA Mutants

**Figure 4:**
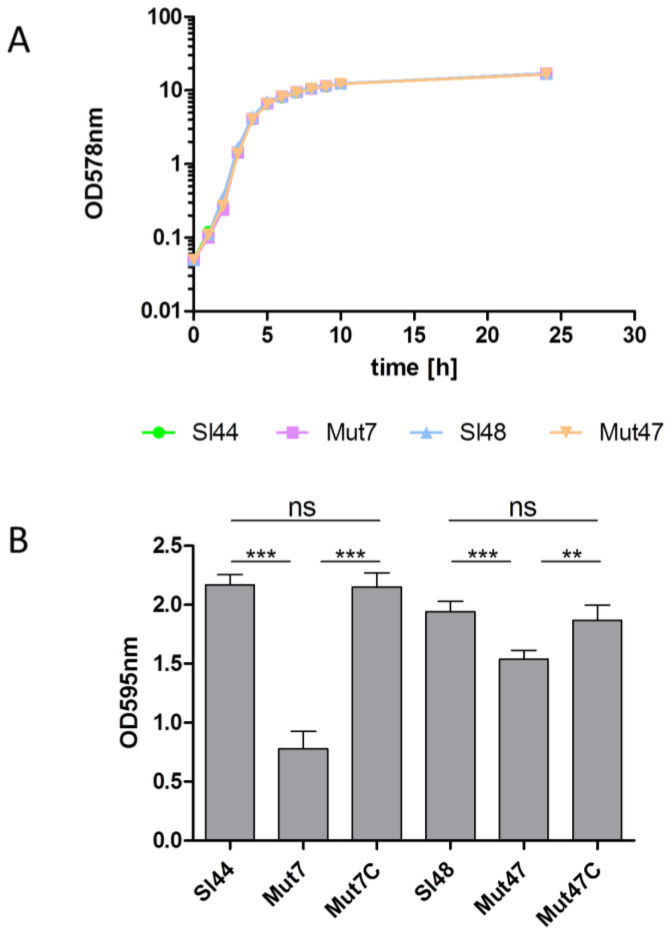
Growth and biofilm formation of *S. lugdunensis* wild-type strains (Sl44, Sl48) and Δ*srtA* mutant strains (Mut7, Mut47). (**A**) Bacterial growth was monitored for 24 h. Bacteria were cultivated in 100 mL BHI in 500 mL flask at 37 °C under permanent agitation. The experiment was done on triplicates. One representative experiment is shown. (**B**) Biofilm-forming capacities of the *S. lugdunensis* wild type (Sl44 and Sl48), Δ*srtA* mutant (Mut7 and Mut47) and complemented mutant (Mut7C and Mut47C) strains were assessed by a quantitative biofilm assay performed in microtiter plates applying crystal violet and determination of the OD_595nm_. Results are shown as the mean of five independent experiments with the standard deviation (SD). Statistical analyses were performed using one-way ANOVA with Bonferroni multiple comparisons posttest (** *p* < 0.01; *** *p* < 0.001). ns—not significant.

The parental strains of *S. lugdunensis* Sl44/Sl48 and Δ*srtA* mutants Mut7/Mut47 showed nearly the same growth rate for 24 h when cultivated in 100 mL of BHI in 500 mL flask (Figure 4A). Furthermore, in little cultivation volumes of only 5 mL in 14 mL glass tubes, no difference in growth could be observed (data not shown). Next, we determined the biofilm formation of all strains. *S. lugdunensis* wild type strains and the Δ*srtA* isogenic mutants were grown in BHI, TSB, and were supplemented with or without additional 0.5% (*w*/*v*) glucose. The addition of 0.5% glucose to TSB resulted in a strong biofilm formation but it was not observed in BHI with glucose. Mut7 produced 70% less biofilm in TSB plus glucose compared to the wild type strain Sl44. Mut47 showed only a decrease of around 25% biofilm formation capability in TSB plus glucose compared to the parental strain Sl48. In the complemented mutants Mut47C and Mut47C, the biofilm formation was completely restored to the levels of the wild-type strains Sl44 and Sl48 (Figure 4B).

### 3.7. Recombinant SrtA As Well As Cell Extracts Catalyzes Hydroxylaminolysis

**Figure 5:**
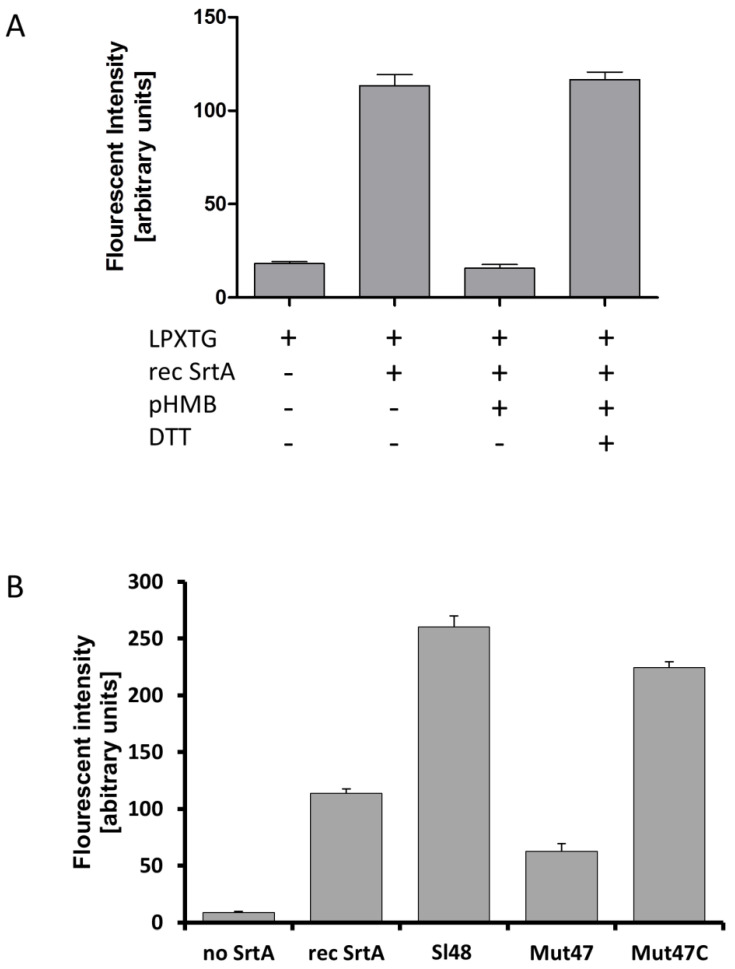
Hydroxylaminolysis of LPXTG peptide by recombinant SrtA (**A**) and whole *S. lugdunensis* cells (**B**). (**A**) Recombinant SrtA was incubated with the sorting substrate Dabcyl-QALPETGEE-Edans (LPXTG), and peptide cleavage was monitored as an increase in fluorescence. The reactions were influenced by the addition of pHMB (inhibitor of sortase activity) and by DTT. Presence and absence of the respective substance is shown as “+” or “-“, respectively. (**B**) Same assay using the LPXTG substrate, recombinant SrtA, *S. lugdunensis* cell extracts from the wild-type strain Sl48, Δ*srtA* mutant Mut47 and complemented Mut47C. All results are shown as the mean of three independent experiments with the standard deviation (SD).

To verify whether our recombinant SrtA as well as native SrtA of cell extracts still had catalytic activities, we incubated them with LPXTG fluorescent peptides (DABCYL-LPETG-EDANS) and CaCl_2_. An increase in fluorescence is observed when cleavage of DABCYL-LPETG-EDANS peptide separates the fluorophore from Dabcyl which in turn confirmed the well described catalytic mechanism of the sortase. First, we tested the catalytic activity of the recombinant sortase SrtA from *S. lugdunensis* in this experimental setup. We observed an increase in fluorescence caused by SrtA which confirmed the cleavage of the DABCYL-LPETG-EDANS peptide (Figure 5A). As hydroxylaminolysis of LPXTG peptides depends on the sulfhydryl of the sortase, an addition of the known sortase inhibitor pHMB at least strongly abolished enzymatic activity of SrtA. However, the enzymatic activity was restored when DTT was added. We next tested cell lysates containing the native SrtA of the wild type strain Sl48, the mutant Mut47, the complemented mutant Mut47C and compare them with the recombinant SrtA in one experiment (Figure 5B). We observed an increase in fluorescence intensity when cell extracts of Sl48, Mut47C and the recombinant SrtA were incubated with the LPXTG peptide in presence of CaCl_2_. As expected, only low activity was measured for cell extracts of the mutant strain Mut47 and the control (Figure 5B). Comparable results were found in cell extracts of the Sl44 wild type, the SrtA deficient mutant Mut7, and the Mut7C complemented mutant strains.

### 3.8. Agglutination Test and Adherence of ΔsrtA Mutants to Immobilized Fibronectin (Fn), Fibrinogen (Fg), and Vitronectin (Vn)

**Figure 6:**
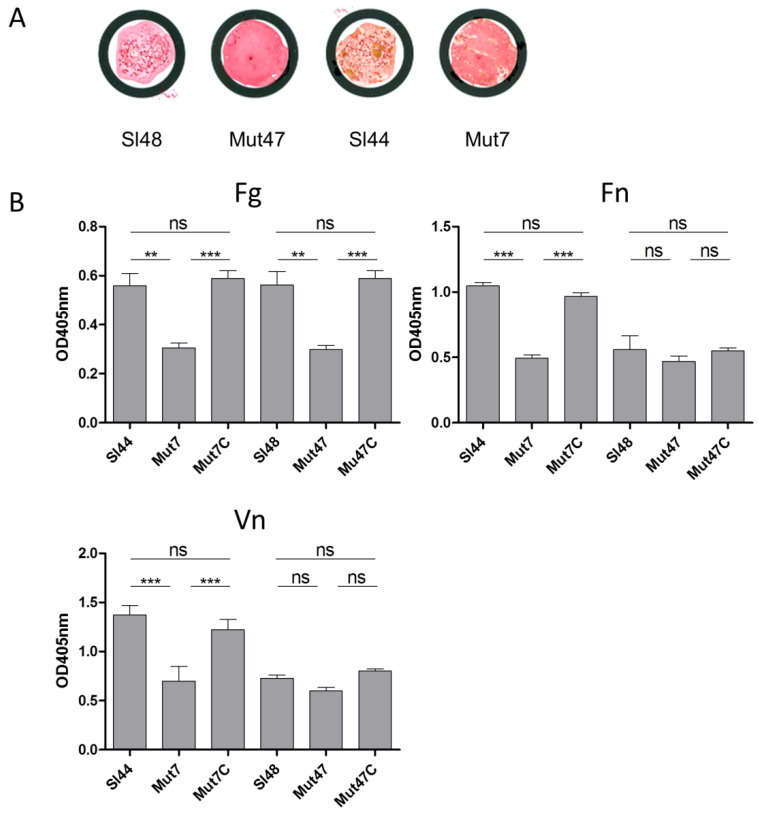
Pastorex staph plus agglutination test and binding of *S. lugdunensis* strains to ECM proteins. (**A**): Pastorex staph plus test of *S. lugdunensis* wild-type strains (Sl44, Sl48) and Δ*srtA* mutant strains (Mut7, Mut47) grown overnight on blood agar plates. Material was mixed in one drop of Pastorex reagent on a Pastorex disposable card. Results were recorded as positive on visual agglutination of wild-type strains and as negative for both mutants showing no agglutination. (**B**): Binding of *S. lugdunensis* wild-type strains (Sl44 and Sl48), Δ*srtA* mutants (Mut7 and Mut47), and complemented mutant strains (Mut7C and Mut47C) to immobilized fibrinogen (Fg), fibronectin (Fn), and vitronectin (Vn) assessed by ELISA adherence assays. Results are shown as the mean of four independent experiments with the standard deviation (SD). Statistical analyses were performed using one-way ANOVA with Bonferroni multiple comparisons posttest (** *p* < 0.01; *** *p* < 0.001). ns—not significant.

Clumping factor (ClfA) of *S. aureus* is a SrtA substrate and constitutes an important virulence factor. It binds to the C-terminus of the γ-chain of fibrinogen and allowed the adhesion to different eukaryotic cell types. Besides, the ClfA is used as an antigen for rapid diagnostic identification based on latex-agglutination test systems. *S. lugdunensis* also possesses a clumping factor (Fbl) with an amino acid identity of 58% to ClfA of *S. aureus*. Hence, we applied the Pastorex Staph Plus (Sanofi Diagnostic Pasteur, S.A., Marnes la Coquette, France) rapid agglutination test for simultaneous detection of the fibrinogen affinity antigen (clumping factor) combined with the detection of protein A and capsular polysaccharides of *S. aureus*. The WT strains showed positive agglutination reactions, but the Δ*srtA* strains did not reveal any agglutination (Figure 6A). It suggested the absence of Fbl in mutant strains, but we verified this result and measured the adherence of the Δ*srtA* mutants to different ECM proteins via ELISA assays. All strains were investigated for binding to immobilized Fn, Fg, and Vn. Compared to wild-type strain Sl44, the adherence of the Δ*srtA* mutant Mut7 was significantly decreased to immobilized Fg, Fn, and Vn (Figure 6B). The binding of complemented mutant Mut7C was indistinguishable to the parent level binding capacity. For the mutant strain Mut47, the results differed a little bit. We observed only a strong decrease in binding Fg, but the binding to Fn- and Vn-coated surfaces of Mut47 decreased less and not to a significant extent. However, binding of complemented mutant Mut47C to Fg, Fn and Vn was restored to the same extent as the wild type strain Sl48 (Figure 6B).

### 3.9. Sortase A Inhibition Resulted in Decreased Biofilm Formation and Binding to Fg and Fn

**Figure 7:**
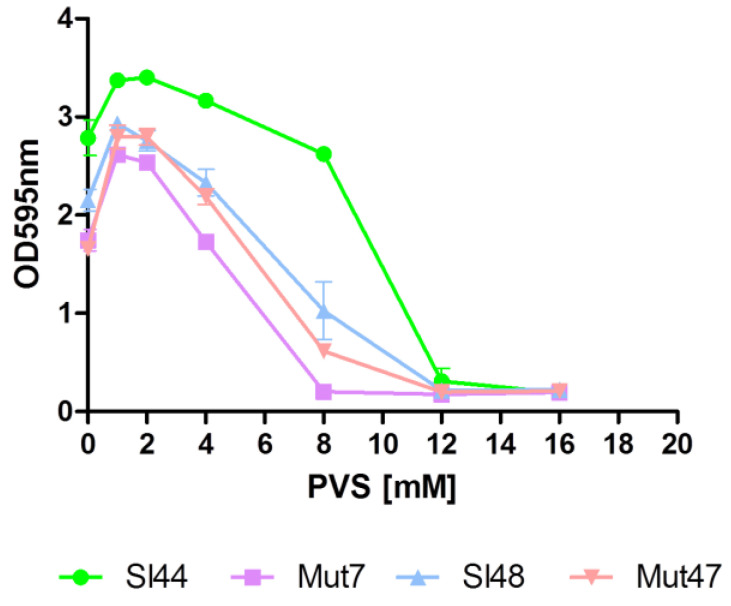
Effect of the sortase A inhibitor phenyl vinyl sulfone (PVS) on biofilm formation. The minimum inhibitory concentrations (MIC) of the sortase inhibitor PVS of *S. lugdunensis* wild type (SL44 and Sl48), Δ*srtA* mutant (Mut7 and Mut47) were found about 12 mM. Data are presented as mean adsorptions of triplicate determinations. Single representative experiments out of three are presented. Error bars show standard deviations (SD).

The function of sortase A could be blocked by different classes of sortase A inhibitors like berberine chloride (BBCl), phenyl vinyl sulfone (PVS) and pHMB as shown before in Section 3.7 [41]. In our study, we used the well-known SrtA blocking reagent PVS. Our experiments with PVS showed MIC’s of about 8-12 mM for strains Sl48, Sl44 and the corresponding Δ*srtA* mutants (Figure 7).

**Figure 8:**
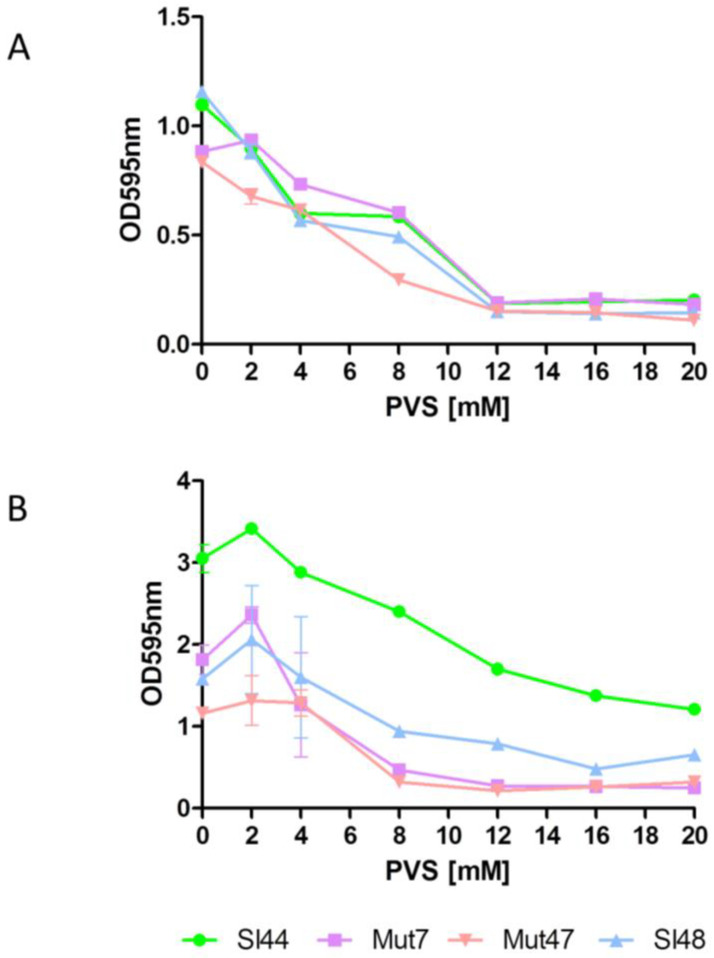
Effect of PVS on binding of *S. lugdunensis* to Fn- (**A**) or Fg- (**B**) coated wells. Overnight cultures were diluted to an OD_595_ of 0.01. 180 µL of diluted culture were mixed with 20 µL of concentrated phenyl vinyl sulfone (PVS) to the respective concentrations in a microtiter plate well. Plates were incubated at room temperature for 1 h. Adherence was determined in a Biorad reader at absorbance of 595 nm. Data are presented as mean adsorptions of triplicate determinations. Single representative experiments out of three are presented. Error bars show standard deviations (SD).

The sortase A inhibition by PVS appeared to be highly effective because at 8 mM concentration we observed a reduction in biofilm formation of strains Sl48 and Sl44, but also for the corresponding Δ*srtA* mutants. We further determined the effect of PVS on the binding capability of *S. lugdunensis* to Fn- and Fg-coated surfaces (Figure 8). A PVS treatment decreased the ability of *S. lugdunensis* to adhere to Fn- and Fg-coated surfaces. PVS at a concentration of 12 mM abolishes the binding to Fn of the parental strains Sl48 and Sl44 and the Δ*srtA* mutants Mut47 and Mut7. The effective PVS concentration to stop binding to Fg is about 16–20 mM (Figure 8).

### 3.10. Hydroxylamine HCl Treatment Decrease Binding to Immobilized Fg and Fn

**Figure 9:**
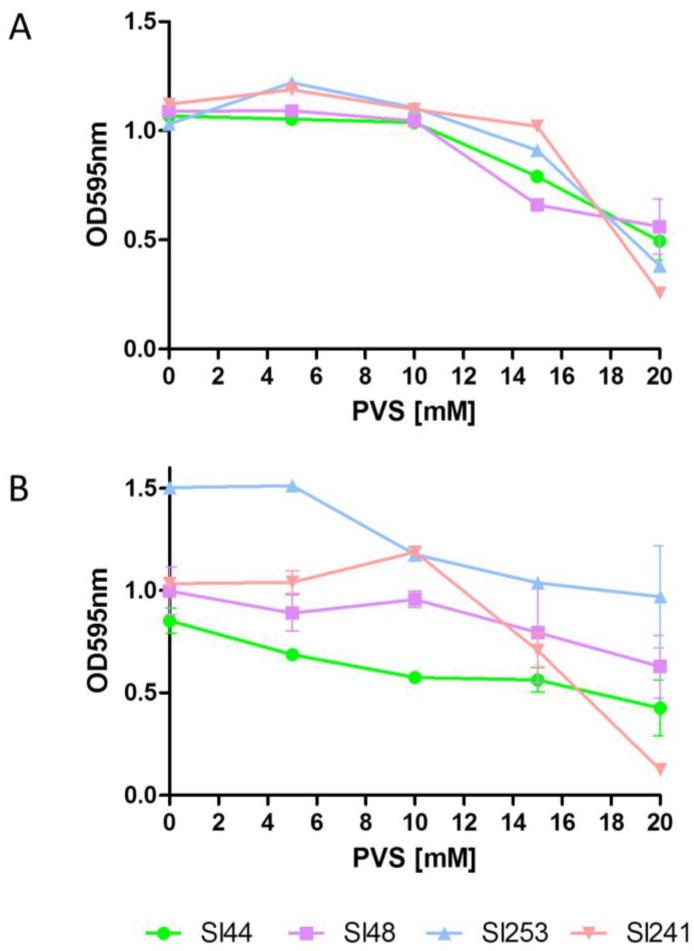
Effect of hydroxylamine HCl on binding of *S. lugdunensis* wild type strains to Fn (**A**) or Fg (**B**) coated wells. Overnight cultures were diluted to an OD_595_ of 0.01. 180 µL of diluted culture were mixed with 20 µL of concentrated hydroxylamine hydrochloride to the respective concentrations in a microtiter plate well. Plates were incubated at room temperature for 1 h. Adherence was determined in a Biorad reader at absorbance of 595 nm. Data are presented as mean adsorptions of triplicate determinations. Single representative experiments out of three are presented. Error bars show standard deviations (SD).

A hydroxylamine treatment causes a formation of the C-terminal threonine hydroxamate of surface proteins, which are thereby released into the culture medium (17). Therefore, a hydroxylamine exposure of *S. lugdunensis* should result in a decreased adherence to Fg, Fn or other eukaryotic proteins. Here, we incubated different wild type strains (Sl44, Sl48, Sl253 and Sl241) with different concentrations of hydroxalamine HCL and found that 20 mM hydroxylamine HCl tendentially reduced the ability to adhere to Fn (Figure 9A). The reduction of Fg adherence was much lower than that observed for adherence to Fn (Figure 9B). In addition, we observed strain specific differences in binding Fg and Fn. 

### 3.11. Adherence and Invasion to Eucaryotic Cell Lines

**Figure 10:**
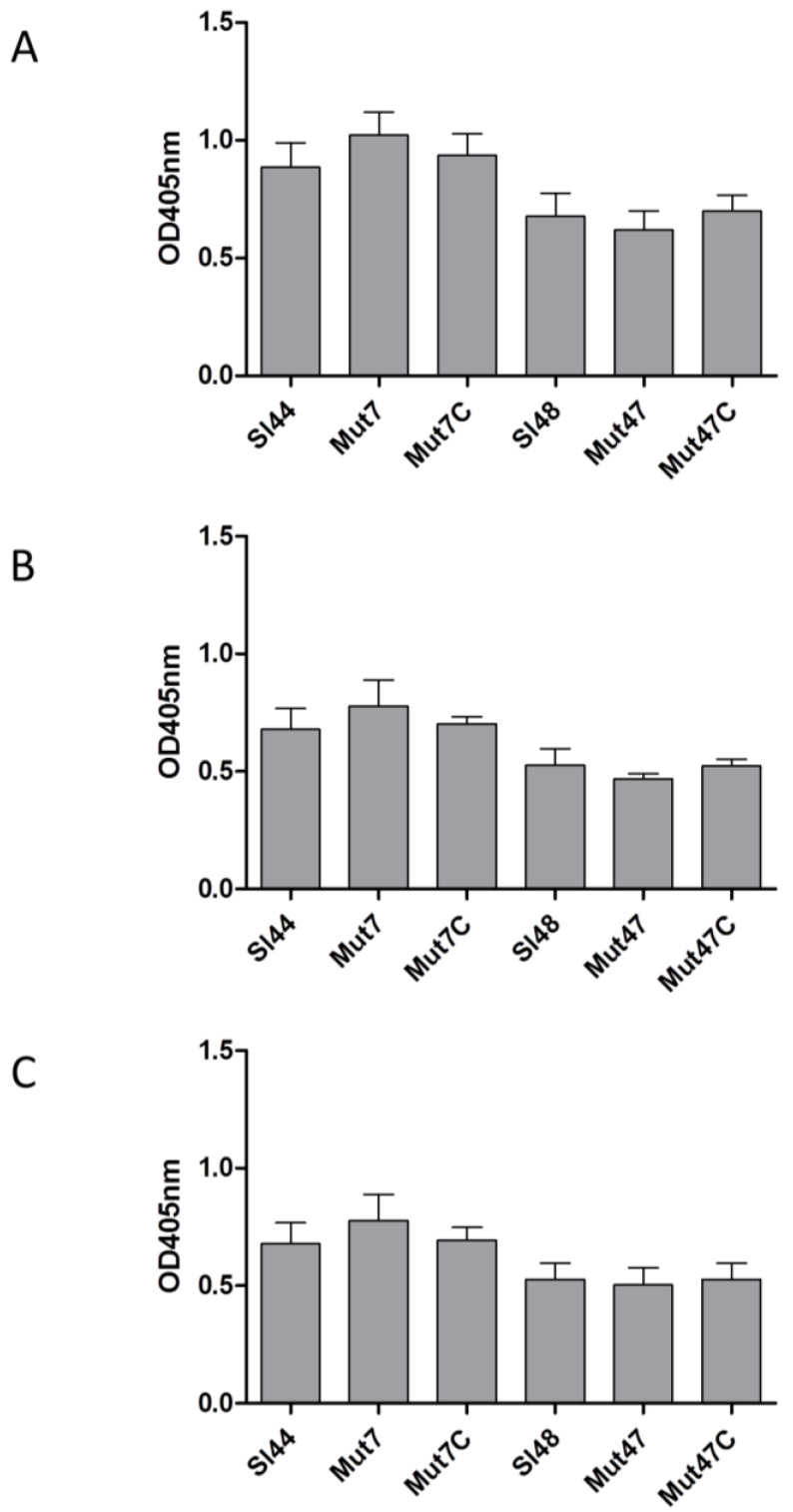
Adherence of *S. lugdunensis* wild-type (Sl44 and Sl48), Δ*srtA* mutant (Mut7 and Mut47) and complemented mutant (Mut7C and Mut47C) strains to immobilized human host cells ((**A**): endothelial cell line EA.hy926, (**B**): fetal lung A549 fibroblasts and (**C**): urinary bladder carcinoma cell line 5637 (ATCC HTB-9™)) assessed by ELISA adherence assays. Results are shown as the mean of at three independent experiments with the standard deviation (SD). Statistical analyses were performed using one-way ANOVA with Bonferroni multiple comparisons posttest, but the differences were not significant.

LPXTG motif cell surface proteins are important for the adherence of staphylococci to eukaryotic cells. As shown above, Δ*srtA* mutants showed decreased adherence to different ECM proteins. Therefore we tested the adherence of the wild type, mutant, and complemented strains to three different eukaryotic cell lines. Surprisingly, the Δ*srtA* mutant Mut47 showed only slight reduced adherence to confluent epithelial cells, fibroblast, and 5637 cells. However, another Δ*srtA* mutant Mut7 bound more to the above mention three cell lines. However, these differences were not statistically significant (Figure 10). 

**Figure 11:**
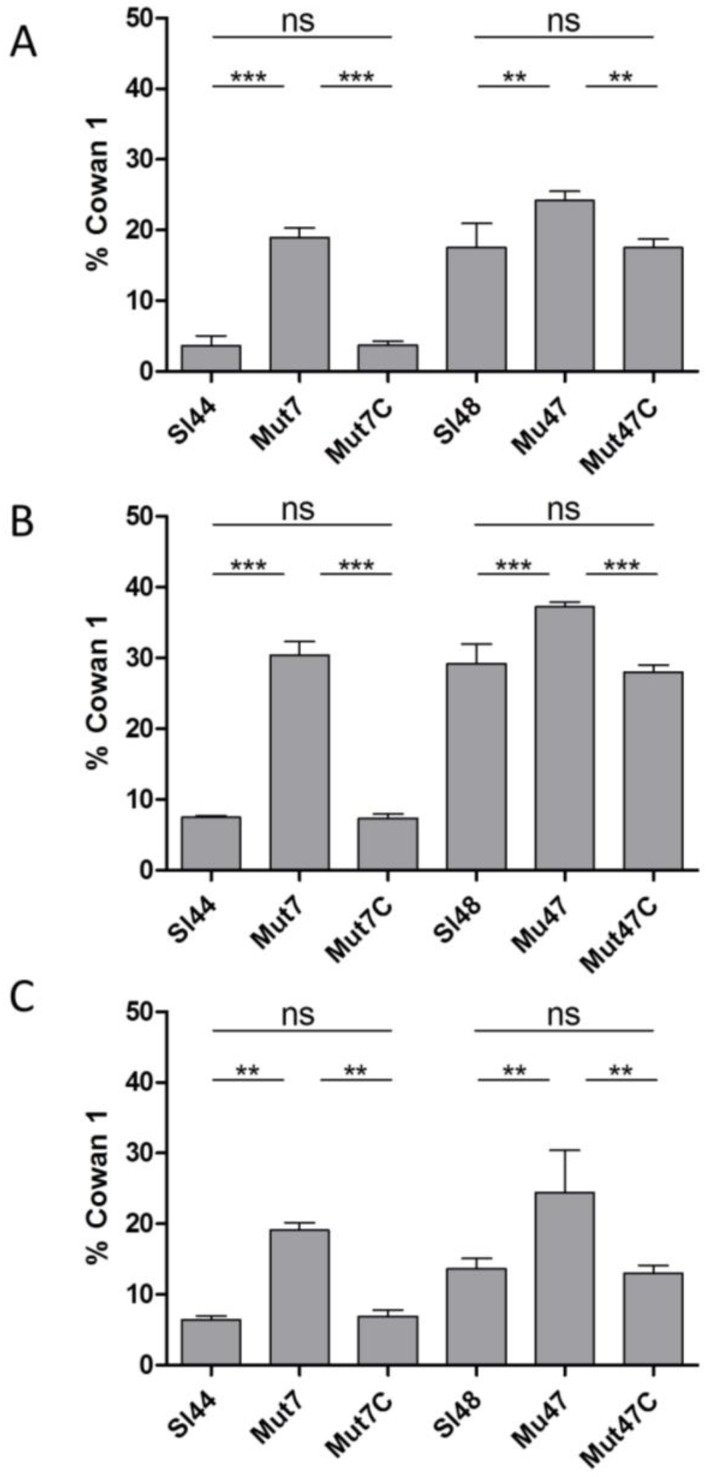
Internalization of FITC-labelled *S. lugdunensis* wild-type (Sl44 and Sl48), Δ*srtA* mutant (Mut7 and Mut47) and complemented mutant (Mut7C and Mut47C) strains by human host cells ((**A**): endothelial cell line EA.hy926, (**B**): fetal lung A549 fibroblasts and (**C**): urinary bladder carcinoma cell line 5637 (ATCC HTB-9™)) assessed by flow cytometry and computed in relation to *S. aureus* Cowan 1. Results are shown as the mean of three independent experiments with the standard deviation (SD). Statistical analyses were performed using one-way ANOVA with Bonferroni multiple comparisons posttest (** *p* < 0.01; *** *p* < 0.001). ns—not significant.

Next, we analyzed the invasion to the same eukaryotic cell lines. In experiments with FITC-labeled wild type and mutant strains, we observed a significant increase in invasion of both mutant strains to 5637 cells, Ea.hy926, and fibroblast cells (Figure 11). In addition, we observed that the Mut7 strain reached same invasion levels as the other mutant Mut47, but the wild type Sl44 revealed a comparable low capability to invade these cell lines. However, complemented strains showed the same invasion behavior as the respective wild type strains.

### 3.12. SrtA Influence the Gene Expression of Further Adhesins

As shown for Δ*srtA* mutants of other species [42], we finally measured the influence of a *srtA* gene deletion on the gene expression of two further LPXTG proteins. Using real-time PCR, expression of specific mRNA for the LPXTG-bearing adhesin proteins Fbl and vWbF were compared in wild type strains Sl44, Sl48 and their respective Δ*srtA* mutants Mut7 and Mut47. The RT-PCR analyses of *fbl* and *vwbF* genes showed a modest up-regulation of about 2 fold in both Δ*srtA* mutant backgrounds (Table 4), verifying results observed for sortase mutants of other species [42].

## 4. Discussion

The LPXTG-anchored proteins are covalently anchored to the cell surface of many bacteria and play key roles as virulence factors for the establishment of bacterial infections [43]. The executive enzyme, the sortase, is essential for the functional assembly of cell surface virulence factors and, hence, important for the pathogenesis of staphylococci and in particular *S. aureus* [22,44]. Mutations in the *srtA* gene result in non-anchoring of 19 surface proteins to the cell envelope [23]. Consequently, *S. aureus* sortase mutants are defective in assembling surface proteins and are highly attenuated in the pathogenesis shown in animal infection studies [23]. However, only a few reports are available on the properties of *S. lugdunensis* in terms of interaction with ECM molecules or host tissues, as well as on its possession of the LPXTG-anchored proteins and secretable expanded repertoire adhesive molecules (SERAMs). *S. lugdunensis* strains were shown to bind to varying extents to collagen, fibronectin, vitronectin, laminin, fibrinogen, thrombospondin, plasminogen, and human IgG [45]. A comparative bioinformatic analysis of publicly available *S. lugdunensis* genomes identified the presence of 11 sortase A dependent proteins (Table 3) [39]. In the published genome sequence of *S. lugdunensis* strain N920143, Heilbronner et al. (2011) identified *fbl*, *vWbF*, *isdB*, *isdJ* and seven more genes coding for proteins with a LPXTG motif [39]. In the present study, we report the characterization of *S. lugdunensis* housekeeping SrtA and its role in LPXTG motif proteins’ cell wall anchoring. To address the role of SrtA to adhere to different ECMs and cell lines, stable and defined Δ*srtA* mutants of *S. lugdunensis* were generated by allelic replacement as described before and respective complemented mutant strains were generated to exclude any polar effects [30]. 

The SrtA from four *S. lugdunensis* strains (N920143, HKU09-01, M23590, and VCU139) showed identity of 76-77% to the SrtA of *S. aureus* strain Newman, and a very high identity of 98-100% among themselves. Specific residues identified in the region corresponding to the described calcium binding cleft in the *S. aureus* SrtA were also highly conserved in the amino acid sequence of the *S. lugdunensis* SrtA. In our study, the recombinant SrtA as well as whole cell extracts of *S. lugdunensis* could catalyze the hydrolysis of a fluorescent quenched polypeptide carrying the LPXTG motif, which is characteristic of sortases of other species (Figure 5) [16,23]. In addition, it was observed that the *S. lugdunensis* SrtA activity also required calcium ions for hydroxyaminolysis of LPXTG peptide, as reported previously for SrtA of *S. aureus* [40]. Therefore, we propose a quite similar catalytic mechanism as shown for SrtA from *S. aureus* [16,17]. These assumption was approved by further phenotypic analyzes showing an essential role for SrtA in anchoring LPXTG proteins, as Δ*srtA* mutants differ in their cell surface proteome from that of the wild type strains, showing the absence of several proteins in the Δ*srtA* mutants (Figure 3). It’s therefore not surprising that we found a reduced biofilm formation and a reduced adherence to Fn, Fg, and Vn in the Δ*srtA* mutants (Figure 4B and Figure 6). Similar results were shown for *S. aureus* as the inhibition of SrtA activity caused loss of binding to Fn, Fg, and IgG and reduced biofilm formation [46,47]. Furthermore, these results are consistent with our observations that the Δ*srtA* mutants showed no agglutination in the Pastorex test.

The presence of an active sortase enzyme in *S. aureus* was found essential for the adherence to host eukaryotic cell [46]. In a previous study, the Δ*srtA* mutants of *S. aureus* and *Lactococcus salivarius* showed significant reductions in adhesion to different epithelial cell lines compared to the WT strain [48,49]. Here in this report, the Δ*srtA* mutant Mut47 showed only slightly reduced adherence to confluent epithelial cells, fibroblast, and 5637 cells, whereas another Δ*srtA* mutant Mut7 bound to above mention cell lines (Figure 10). Obviously, the differences were not statistically significant, which was surprising because formerly we observed significant lower adherences to Fn, Fg, and Vn. As shown previously, the autolysin AtlL of *S. lugdunensis* was identified as a major adhesin being crucially involved in the internalization process to cells [47]. Here, we showed a fairly strong imbalance in the proteome of the cell surface compartment in the mutant strains. These probably make AtlL more available to the cell surface, which might compensate for the loss of other LPXTG adhesins in case of Mut47 and eventually enhance, to a limited extent, the binding of Mut7 to eukaryotic cells. On the other hand, we cannot exclude the absence of the respective LPXTG counterpart proteins on the cell surfaces of the eukaryotic cell lines under our experimental conditions. This rather would lead to indistinguishable results between the adherence of wild types and Δ*srtA* mutant to cell lines, similar to what we have observed here. 

We further observed that SrtA is not only involved in the covalent binding of LPXTG motif proteins to cell wall, but also influenced the transcriptional regulation of at least two adhesin genes *fbl* and *vWbF*. RT-PCR results showed that expression of *fbl* and *vWbI* genes were upregulated at least 2 fold in the Δ*srtA* mutants relative to their wild type strains (Table 4). This is in agreement with the previous finding that mutations in the *srtA* gene result in the upregulated expression of cell surface proteins in an oral *Streptococcus* strain [42]. Nobbs et al. (2007) reported a significant upregulation of the expression of adhesin genes like *sspA/B*, *cshA/B*, and *fbpA* in Δ*srtA* deficient mutants relative to their wild type strains of *S. gordonii* [50]. Moreover, Hall et al. (2019) recently described a quality control mechanism that monitors the processing of LPXTG adhesins by SrtA via measuring the left C-terminal cleaved LPXTG proteins (C-peps) which stayed membrane located and are recognized by a previously uncharacterized intramembrane two-component system (TCS) [51]. Prevention of C-peps generation de-repressed this TCS and resulted in increased expression of further adhesins compensating for the loss of LXPTG adhesins [51]. Since TCS is conserved in streptococci but not in staphylococci, the same C-pep-driven regulatory circuit is unlikely in *S. lugdunensis*, but similar regulation processes cannot be ruled out and might be an explanation for the upregulation of *fbl* and *vWbF*. Thus, the comparable high adherence to host cells of Δ*srtA* mutants might also be a result of the upregulation of other adhesins in the mutant strains and is object of future research. However, detailed in vitro infection studies are necessary to understand the impact of the single LPXTG proteins of *S. lugdunensis* to the respective adherence mechanisms.

A sudden inhibition of the SrtA activity could disrupt the pathogenesis of bacterial infections [22]. The surface-protein anchoring is sensitive to sulfhydryl-modifying reagents like methanethiosulfonates, berberine chloride, (Z)-3-(2,5-dimethoxyphenyl)-2-(4-methoxyphenyl)acrylonitrile or PVS under in vitro conditions [17,42,52]. Here, the SrtA inhibitor PVS reduced the biofilm formation and also reduced the adherence to Fg and Fn (Figure 7 and Figure 8). Further, a hydroxylamine HCl treatment, releasing LPXTG proteins from the cell wall, was found to greatly reduce the adherence of four wild type strains of *S. lugdunensis* to immobilized Fg and Fn (Figure 9). This clearly showed the important role of the LPXTG proteins in *S. lugdunensis* for the binding to some ECM proteins, and confirmed the data observed for *S. aureus* [23,53]. The inhibition of the sortase activity may provide a novel approach for the treatment of infections with staphylococci and a development of such inhibitors could complement the current dependence on antibiotics. Therefore, SrtA is an attractive target to attenuate virulence and hamper infections. In addition, the Δ*srtA* mutant of *S. lugdunensis* showed significant defects in virulence including reduced bacteremia, reduced bacterial spreading to the kidneys and reduced size/density of endocardial vegetations [24]. This highlights the importance of LPXTG-anchored proteins in the pathogenesis of this germ. These and our results suggest that several surface proteins probably act in concert to promote the adhesion to different host structures and enable a survival in the host [24]. Sortase A mutants of *S. aureus* displayed strongly reduced virulence in various infection models including experimental sepsis, highlighting the impact of properly displayed surface proteins during infection [54,55]. Therefore, the Δ*srtA* mutants were recently discussed as vaccine candidates due to the attenuated but still immune-stimulating phenotype [56]. 

In conclusions, SrtA was found as the housekeeping sortase of *S. lugdunensis* like in other Gram-positive bacteria and in particular in *S. aureus*. Disrupting the presentation of surface proteins by gene deletion or blocking the activity of SrtA via different SrtA inhibitors could therefore effectively cause a reduction in binding to ECM proteins and could probably disrupt the pathogenesis of bacterial infections and promote bacterial clearance by the host. Although some strain-specific differences may exist, SrtA could be an attractive target to attenuate the virulence of *S. lugdunensis*.

## Figures and Tables

**Table 1:** Bacterial strains used in this study.

Strains	Relevant Genotype or Plasmid	Properties	Reference or Source
*S. lugdunensis* strains			
Sl48		Clinical isolate	Germany ^b^
Sl44		Clinical isolate	Germany ^b^
Mut7	Sl48 *srtA::EmR*	Sl48 deficient in sortase-A	This study
Mut47	Sl44 *srtA::EmR*	Sl44 deficient in sortase-A	This study
SL241		Clinical isolate	Germany ^b^
SL253		Clinical isolate	Germany ^b^
*S. aureus* strain			
*S. aureus* Cowan 1 (ATCC 12598)		Reference isolate from septic arthritis	ATCC
*E. coli* strains			
DH5α	*supE44ΔlacU169 (*ϕ80 *lacZΔM15) hsdR17 recA1 end A1 gyrA96 thi-1 relA1*	Cloning host	Stratagene
TG1	*supE hsd* *Δ5 thi* *Δ(lac-proAB) F’(traD36 proAB^+^ lacI^q^ lacZ* *ΔM15)*	Cloning host	Stratagene
DH5α (pBT37)	pBT9*atlL::Em^R^*	Shuttle vector pBT9 containing *atlL::Em^R^*	This study
Eukaryotic strains			
EA.hy 926 cells			[25]
A549 fibroblast			[26]
Human bladder carcinoma cell line 5637			[27]

^b^ kindly provided by F. Szabados and S. Gatermann (Bochum, Germany).

**Table 2:** Primer sequences used in this study.

Primer	Sequence (5′–3′)	Reference
srtA1FH	AAAAAGCTTTAAGAAAGCTAAAAAAATGACATAGTTG	This study
srtA1RE	AAAGAATTCCTCCAATAATGGTCATCAATTGGTTTGTCC	This study
srtA2FE	AAGAATTCTATTTATAGCAGAACAGATTAAATAATTGTAG	This study
srtA2RB	AAAGGATCCCATCTGAGTCAA GACTACTAGCAAGTGG	This study
Ery-EF,	ATATATCGATTAGGGACCTCTTTAGC	[28]
Ery-ER	ATATATCGATATCATGAGTATTGTCCG	[28]
SrtA1FH	AAAAAGCTTTAAGAAAGCTAAAAAAATGACATAGTTG	This study
SrtA2RB	AAAGGATCCCATCTGAGTCAAGACTACTAGCAAGTGG	This study
srtAF	CTCGGATCCAAACCTCATATTGATAGTTATTTACATGAC	This study
srtAR	CTCGGTACCTTATTTAATCTGTTCTGCTATAAATATTTTACGC	This study
RTFblF	GAAGCAACAACGCAGAACAA	[38]
RTFblR	TGCTTGTGCCTCGCTATTTA	[38]
RT16SF	CAGCTCGTGTCGTGAGATGT	[38]
RT16SR	TAGCACGTGTGTAGCCCAAA	[38]
RTvWbF	GGACCAGGTGAAGGTGATGT	This study
RTvWbR	GCCGCTGATTTTCGTGTAAT	This study

**Table 3:** Known *S. lugdunensis* putative sortase-A mediated cell surface proteins with relevant properties [39].

Genetic Identifiers (GN)	Annotation	Cleavage Motif	Size (aa)	Predicted Protein Size (kDa)	NCBI BLAST Hit (Protein ^1^, Strain ^2^, Length ^3^)
SLUG_00890 SLGD_00061	IsdB	LPATG	690	76.9	Surface protein SasI, HKU09-01
SLUG_00930 SLGD_00065 SasE	IsdJ	LPNTG	646	71.5	Cell surface protein IsdA, HKU09-01 LPXTG cell wall surface anchor protein, M23590
SLUG_02990 SLGD_00301	SlsF	LPASG	659	73.4	Predicted cell-wall-anchored protein SasF, HKU09-01
SLUG_03480 SLGD_00351	SlsA	LPDTG	1930	207.3	Cell wall associated biofilm protein, HKU09-01, 3799
SLUG_03490 SEVCU139_1800 SLGD_00352	SlsD ^4^	LPATG	1619	175.8	Putative serine-aspartate repeat protein F, VCU139, 2190 Putative uncharacterized protein, HKU09-01, 1136
SLUG_03850 SLGD_00389 HMPREF0790_1688	Slsc	LPETG	190	21	LPXTG protein, HKU09-01 Cell wall surface anchor family protein, M23590, 196
SLUG_04710 SEVCU139_1680 SLGD_00473	SlsE	LPETG	3459	364	Gram-positive signal peptide protein, VCU139, 2988 Hypothetical membrane protein, HKU09-01, 3232
SLUG_04760 SLGD_00478	SlsB	LPNTG	277	30.6	Putative uncharacterized protein, HKU09-01
SLUG_22400 SLGD_02322 bca PE	SlsG	LPDTG	2079	222.1	Putative uncharacterized protein, HKU09-01, 2886 C protein alpha-antigen, VCU139, 2031
SLUG_16350 HMPREF0790_0533 SLGD_01633	Fbl	LPKTG	881	94.2	Clumping factor A, M23590, 857 Clumping factor A (fragment), VCU139, 688 Methicillin-resistant surface protein, HKU09-01, 701
SLUG_23290 SLGD_02429	vWbF	LPETG	1869	209.4	Von Willebrand factor-binding protein, HKU09-01, 2194

^1^ Different designation as given in annotation column. ^2^ Strain different from N920143. Annotation, cleavage motif, size (aa), and predicted protein size (kDa) columns are based on strain N920143. ^3^ Length if different from size (aa) column. ^4^ The *slsD* contains a nonsense codon located just 5′ to the region encoding LPXTG.

**Table 4:** Real-time quantification of *fbl* and *vWbF*genes.

Strain	fbl ^a^	vWbF ^a^
Mut47 vs. Sl48	1.88 ± 0.12	2.35 ± 0.25
Mut7 vs. Sl44	1.99 ± 0.18	1.68 ± 0.12

^a^ Relative levels of *fbl* and *vWbF* specific RNA in *S. lugdunensis srtA* deletion mutants were compared to wild-type strains. The fold changes in gene expression of *fbl* and *vWbF* are shown for the Δ*srtA* mutants Mut47 and Mut7 relative to wild type strains Sl48 and Sl44. Above given values represent mean ± SD of three independent experiments performed in triplicate.
